# Vein of Marshall Ethanol Infusion for AF Ablation; A Review

**DOI:** 10.3390/jcm13082438

**Published:** 2024-04-22

**Authors:** Louisa O’Neill, Benjamin De Becker, Maarten De Smet, Clara Francois, Rene Tavernier, Mattias Duytschaever, Jean-Benoit Le Polain De Waroux, Sebastien Knecht

**Affiliations:** 1 Department of Cardiology, AZ Sint-Jan Hospital, 8000 Bruges, Belgium; benjamin.debecker@azsintjan.be (B.D.B.); sebastien.knecht@azsintjan.be (S.K.); 2 Department of Cardiology, Blackrock Clinic, A94 E4X7 Dublin, Ireland; 3 King’s College London, St. Thomas’ Hospital, London SE1 9NH, UK

**Keywords:** atrial fibrillation, Vein of Marshall, ethanol infusion, mitral isthmus ablation

## Abstract

The outcomes of persistent atrial fibrillation (AF) ablation are modest with various adjunctive strategies beyond pulmonary vein isolation (PVI) yielding largely disappointing results in randomised controlled trials. Linear ablation is a commonly employed adjunct strategy but is limited by difficulty in achieving durable bidirectional block, particularly at the mitral isthmus. Epicardial connections play a role in AF initiation and perpetuation. The ligament of Marshall has been implicated as a source of AF triggers and is known to harbour sympathetic and parasympathetic nerve fibres that contribute to AF perpetuation. Ethanol infusion into the Vein of Marshall, a remnant of the superior vena cava and key component of the ligament of Marshall, may eliminate these AF triggers and can facilitate the ease of obtaining durable mitral isthmus block. While early trials have demonstrated the potential of Vein of Marshall ‘ethanolisation’ to reduce arrhythmia recurrence after persistent AF ablation, further randomised trials are needed to fully determine the potential long-term outcome benefits afforded by this technique.

## 1. Introduction

Atrial fibrillation (AF) is the most common cardiac arrhythmia globally with a twofold increase in prevalence predicted over the next 50 years, at which point approximately 17.9 million adults will be living with the condition [1]. As a management strategy, catheter ablation has been shown to be superior to anti-arrhythmic drugs and is associated with a significant reduction in AF burden [2,3], a parameter strongly associated with hard clinical endpoints including stroke, cardiovascular hospitalization and mortality [4,5,6]. Pulmonary vein isolation (PVI) alone is an effective treatment in the paroxysmal AF cohort with high single procedure success rates of up to 90% achievable with optimised workflows [7]. Replicating these results in the persistent AF population has been difficult, with single procedure success rates of 50–60% at one year [8,9,10,11,12]. In these patients, progressive remodelling distant to the pulmonary veins has prompted the evaluation of several strategies of adjunct ablation beyond PVI, with relatively disappointing results in randomised trials [9,10,11,12,13,14]. Linear ablation at the left atrial roof and mitral isthmus represents a commonly employed adjunctive strategy for persistent AF. One of its drawbacks, perhaps partly reflecting the underwhelming results in randomised trials, is the difficulty in achieving durable bidirectional block across ablated lines, particularly at the mitral isthmus [9].

The Vein of Marshall (VoM) is an embryological remnant of the superior vena cava that has been has been implicated as a potential source of AF triggers and is known to harbour sympathetic and parasympathetic nerve fibres that play a significant role in the pathogenesis and maintenance of AF [15,16]. Ethanol infusion of this vein during AF ablation may eliminate these triggers and may facilitate mitral isthmus block, given its anatomic location at the mitral isthmus [17], potentially overcoming one of the main challenges with adjunct linear ablation. Promising results have been demonstrated in the Venus randomised trial of persistent AF ablation with significantly higher arrhythmia-free survival demonstrated in those receiving concomitant VoM ‘ethanolisation’ [18]. In this review, we outline the anatomy and technique for ethanolisation of the VoM and provide an overview of the literature detailing its use in atrial fibrillation and tachycardia (AT) ablation.

## 2. Anatomy and Electrophysiology of Vein of Marshall

### 2.1. Anatomical Considerations

The ligament of Marshall (LoM) was first described in 1850 by Marshall [19]. A vestigial fold resulting from embryological regression of the left anterior cardinal vein [20], the LoM traverses the epicardial surface of the left lateral ridge between the left atrial appendage and the left pulmonary veins. Within this recess, the Vein of Marshall or the left atrial oblique vein runs over the posterior left atrium towards the left superior pulmonary vein, and drains into the great cardiac vein at the point where it becomes the coronary sinus (CS) [21]. 

In adults, the LoM comprises the VoM and the Marshall bundle (MB), a muscular band which runs alongside autonomic nerves [22] and can be considered as an electroanatomical pathway linking the left lateral ridge and the muscle sleeve of the CS. Muscular connections are well described in histopathological studies between the LoM and the CS musculature proximally, and the atrial and pulmonary vein myocardium more distally [23]. These connections to the left atrium (LA) are most frequently observed close to the CS and more distally at the left pulmonary veins. An in vivo mapping study demonstrated that the majority of 72 patients studied had multiple MB–LA connections. Furthermore, fractionated MB electrograms noted were recorded during AF in those with multiple connections [24]. These connections may also have a role in atrial electrophysiology in sinus rhythm and were demonstrated to contribute to normal LA activation in a canine mapping study [25].

In addition to the muscle bundles described above, the LoM is richly innervated. In an early canine study in 1972, left cardiac sympathetic stimulation induced an ectopic atrial rhythm from the region of the LoM [26]. A subsequent study of post-mortem human hearts confirmed the presence of sympathetic fibres on tyrosine hydroxylase staining [27], the distribution of which tends to be most prominent distally in the LoM near the LA-pulmonary vein junctions [23]. Conversely, parasympathetic fibres are most dense at the CS junction, becoming less predominant more distally in the LoM [23]. In line with this, in a canine model, a lower voltage threshold for induction of AF using high-frequency-stimulation was seen proximally in the LoM, increasing distally [28]. These effects were inhibited by the administration of esmelol and atropine and highlight a potential LoM mediated, autonomic basis for AF initiation. In a further canine study, attenuation in vagally-mediated ERP shortening was demonstrated post ablation of the LoM, again suggesting the functional relevance of the structure to atrial electrophysiology [16]. The LoM/VoM may also provide a connection between intrathoracic cardiac ganglia and intrinsic cardiac nerves which tend to cluster in ganglia near the pulmonary veins, in particular, the inferior left ganglion is closely aligned with the VoM [20]. A 2014 study demonstrated the elimination of local intrinsic cardiac nerve responses post VoM ethanol infusion and the utility of the VoM as a vascular route for therapies targeting these intrinsic ganglia [29].

### 2.2. Role of VoM in ATA

These richly innervated electroanatomic connections implicate the LoM/VoM as a potential therapeutic target in treatment of atrial fibrillation and atrial tachycardia (AT), with a well-documented role as both a source of AF triggers as well as an important structure for perpetuation of macro re-entrant atrial tachycardia. A computerised mapping study using canine tissue implicated the LoM as a source of spontaneous atrial activity, with ablation of the LoM eliminating this ectopy and resultant degeneration to AF [30]. Furthermore, in humans, rapid discharges from the LoM can initiate paroxysms of AF, with termination of AF demonstrated after ablation at the insertion site of the VoM [31,32]. In addition to AF triggers, the muscle bundles present in the LoM may serve as critical components of macro re-entrant peri-mitral flutter, the most common AT seen post AF ablation [33,34]. A prior optical mapping study demonstrated slow, non-decremental conduction within these bundles, with induction of macro re-entrant AT seen in the setting of conduction block across CS–LA connections [35]. Additionally, a 2019 clinical study of 199 LA tachycardias post AF ablation demonstrated the involvement of the MB network in up to 29% of macro re-entrant ATs as well as in localised re-entry circuits [36]. The same authors have identified several criteria for MB-related perimitral AT including a percentage of total cycle length mapped at <90% and a PPI–TCL <20 ms at the VoM and the left atrial appendage and pulmonary vein ridge [37]. 

## 3. Targeting the LoM and VoM in Atrial Arrhythmia

### 3.1. Conventional Ablation

Elimination of arrhythmogenic MBs may be possible with endocardial RF ablation. The MB lies within 3 mm of the endocardial surface of the left lateral ridge [37] and is closest to the endocardium inferior to the ostium of the left inferior pulmonary vein [38]. Using a CS angiogram to locate the VoM, Hwang et al. have described the technique for ablation MB signals, using a 1.5 Fr quadripolar catheter inserted into the VoM to guide the site of endocardial ablation [32,38]. Ablation at 30 W eliminated MB potentials in >90%, but residual potentials at the ‘thicker’ left atrial appendage/pulmonary vein ridge, can be difficult to fully eliminate endocardially [38]. More recently, Kashimura et al. describe a 70% success rate of MB isolation using RF ablation endocardially and within the CS, guided by a VoM catheter as above [39]. 

### 3.2. VoM Ethanol Infusion

Failure to isolate the MB endocardially relates to anatomic distance from the endocardial surface and the presence of overlying fat. Similarly, difficulty in achieving bidirectional block endocardially at the mitral isthmus is well documented and relates to tissue thickness, anatomic variations and the presence of isthmi and cavities [40,41,42]. As such, ethanol infusion into the VoM, to ablate the area of LA endocardium it drains, was proposed to overcome these challenges and as a more effective method of eliminating MB triggers for AF. In 2009, Valderrabano first outlined the feasibility of cannulation and ethanol delivery to the VoMs of dogs, with elimination of vagally-mediated ERP shortening post infusion [43]. In humans, an early study of 14 patients undergoing PVI demonstrated the communication of the VoM with the underlying LA myocardium and the feasibility of ethanol infusion into the vein with resultant creation of a low voltage area in the region drained by the VoM [44].

## 4. Evidence for Clinical Benefit for VoM/LoM Ablation

### 4.1. Atrial Fibrillation

As mentioned above, small animal and human studies have demonstrated termination of AF with ablation at the LoM/VoM [30,31,32]. During AF ablation, VoM ethanolisation may increase rates of first-pass isolation of the left pulmonary veins and reduce the incidence of acute reconnection, as demonstrated in a recent study by Huang et al. [45]. Regarding long-term outcomes, in recent years VoM ethanolisation has been evaluated as an adjunct strategy in patients undergoing catheter ablation for predominantly persistent AF. A single centre study of 61 patients undergoing repeat ablation for recurrent AF or AT, demonstrated VoM mediated epicardial connections in reconnected left inferior pulmonary veins, with re-isolation demonstrated post VoM ethanolisation, although this was somewhat dependent on VoM anatomy [46]. In this study, 8 of 54 patients with successful VoM ethanolisation had recurrent arrythmia at 6 months (2 with AF and 6 with atrial flutters). A 2019 non-randomised study compared outcomes between patients undergoing AF ablation, predominantly as a redo procedure, with substrate ablation and VoM ethanol infusion, to those undergoing PVI plus substrate ablation and PVI alone [47]. While only 13% of patients in this study received VoM ethanol infusion, on multivariate analysis it was identified as an independent predictor of freedom from recurrent arrhythmia. 

In 2020, Valderrábano et al. reported on outcomes of the VENUS multicentre randomised trial of adjunctive VoM ethanolisation in first-time persistent AF ablation [18]. Patients were randomised to catheter ablation alone (n = 158) versus catheter ablation plus VoM ethanol infusion (n = 185). Successful VoM infusion was performed in 155 of 185 patients. At 6- and 12-month follow-up, a significantly higher freedom from recurrent atrial tachyarrhythmia was observed in patients who underwent VoM ethanol infusion compared to those who had not (*p* = 0.04), with peri-mitral block a significant predictor of post-procedural success [48]. It is important to highlight that rates of arrhythmia-free survival were modest, however (65.2% vs. 53.8%), and the presence of significant additional substrate ablation in both study groups limits the capacity to accurately determine the added value of VoM ethanol infusion. 

A 2022 meta-analysis of six studies, including 1337 patients, addressed the value of adjunct VoM ethanolisation in persistent AF on long-term outcome [49]. The authors reported improved arrhythmia free survival with adjunct VoM ethanolisation versus standard ablation, with a similar safety profile. Interestingly, this study suggested that study sample size may have a positive impact on outcome, findings supported by sub analysis of the Venus trial, suggesting that better long-term outcomes are achievable in high-volume centres, reflecting the learning curve associated with the technique. 

In 2019, Pambrun et al. reported the results of a series of 10 patients undergoing the ‘Marshall Plan’ ablation strategy for persistent AF, a structured end-point driven lesion set consisting of PVI and linear ablation at the mitral isthmus (facilitated by VoM ethanolisation) roof and cavotricuspid isthmus [50]. The lesion set was achieved in all patients and although procedure times were long at an average of 270 min, all patients were free from recurrence at 6 months. Following from this, Pambrun et al. published their ongoing experience with the ‘Marshall Plan’ strategy in 75 consecutive patients with persistent AF [51]. VoM ethanolisation was achieved in 92% and the full lesion set in 91%. At one year, single procedure success rates were 72% without anti arrhythmic drugs and 79% in those with successful VoM ethanolisation. In a further non randomised study in 2021, Liu et al. evaluated the effects of adding VoM ethanolisation to the ‘2C3L’ (two circle, 3 line) ablation strategy consisting of the above-mentioned lesion set (PVI, mitral isthmus, roof and CTI ablation) in 191 persistent AF patients [52]. At 12-month follow-up, significantly more patients undergoing the ‘upgraded’ approach with VoM ethanol infusion were free from recurrence compared to those undergoing the standard lesion set with RF only (87.9 vs. 64.8%, *p* < 0.001).

Two randomised controlled trials evaluating the identical ‘Marshall Plan’ and ‘upgraded 2C3L’ strategies are currently ongoing. Preliminary 10 month results of the single-centre randomised study of the Marshall plan vs. PVI alone in 120 persistent AF patients were presented in 2023 and documented significantly higher success rates in those receiving the Marhsall plan lesion set (87 vs. 70%) [53]; 12-month outcomes are awaited. Additionally, the PROMPT-AF multicentre randomised trial will recruit 498 persistent AF patients to either the upgraded 2C3L approach vs. PVI only, and compare freedom from recurrence at 12 months [54]. The results of these studies will shed additional light on the potential benefit afforded by adjunct VoM in the challenging persistent AF population. 

### 4.2. Mitral Isthmus Ablation

While more data on long-term outcomes in AF are needed, there is no doubt that VoM ethanol infusion facilitates effective mitral isthmus block, perhaps reflecting the greatest additional benefit afforded by the technique. Significantly higher rates of acute mitral isthmus block (98.7% vs. 63.6%) were demonstrated in a comparative study of 262 patients undergoing adjunct VoM vs. RF only ablation, with higher rates of persistent block seen at repeat procedure [55]. Additionally, a 2020 study of 103 patients presenting with perimetral flutter post prior AF ablation demonstrated higher rates of acute mitral isthmus block with a reduction in the duration of RF ablation needed to achieve AT termination and block, further translating into less recurrence on midterm follow-up [56]. These findings were echoed by a 2023 meta-analysis of 9 studies, including 2508 patients that compared adjunct VoM to catheter ablation alone, and reported significantly higher rates of acute bidirectional block at the mitral isthmus (*p* = 0.0007) with lower rates of recurrent AT and AF post blanking period (*p* = 0.008) [17].

A small randomised study by Gillis et al. from 2021 underscored the above and highlighted the importance of the timing of VoM in the sequence of ablation, with a reduction in the amount of RF applications needed to achieve acute block when VoM ethanol infusion was performed as a first step [57]. These findings were further emphasised in a recent study by Du et al., whereby empirical VoM before catheter ablation at the mitral isthmus resulted in a lower endocardial ablation time and greater long-term AT free survival than when it was performed after failure of endocardial ablation to achieve block [58]. 

In addition to facilitating mitral isthmus block, it has been postulated that VoM ethanolisation may also facilitate left atrial posterior wall ablation through elimination of epicardial connections not accessible with endocardial ablation [59]; nevertheless, Ishimura et al. failed to demonstrate an additional benefit for VoM infusion in 417 patients undergoing posterior wall ablation in a recent comparative study [60].

## 5. Technique for VoM Ethanol Infusion

**Figure 1:**
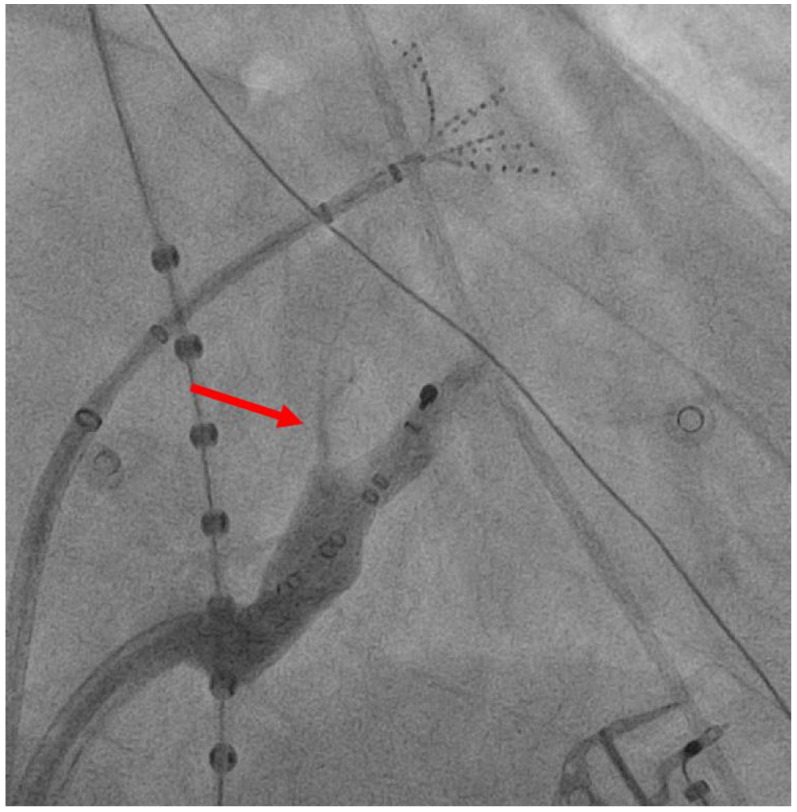
Contrast injection of Vein of Marshall (VoM) (red arrow) through the internal mammary artery (IMA) catheter in the right anterior oblique (RAO) view. Pentaray catheter is visualised in left atrium. Decapolar catheter in coronary sinus. IMA = internal mammary artery, RAO = right anterior oblique.

**Figure 2:**
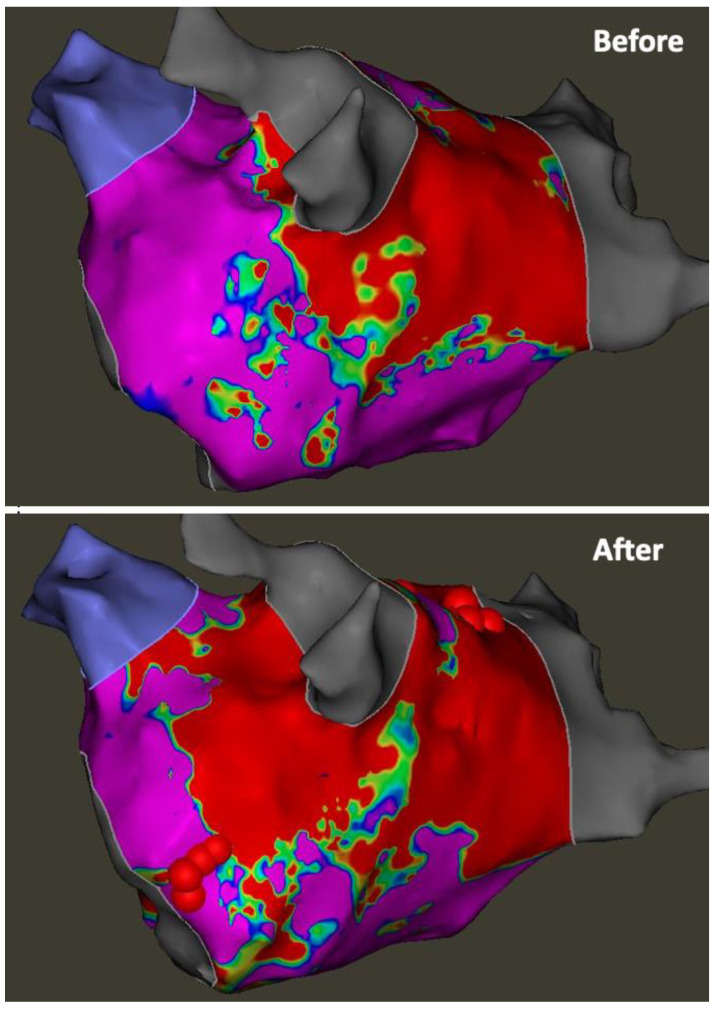
(**Top panel**): voltage map before VoM ethanolisation. (**Bottom panel**): voltage map demonstrating VoM ‘effect’ with new area of confluent scar inferior and anterior to left pulmonary veins. Ablation tags at mitral isthmus indicate the site of endocardial ablation performed to achieve mitral isthmus block post VoM ethanolisation.

**Figure 3:**
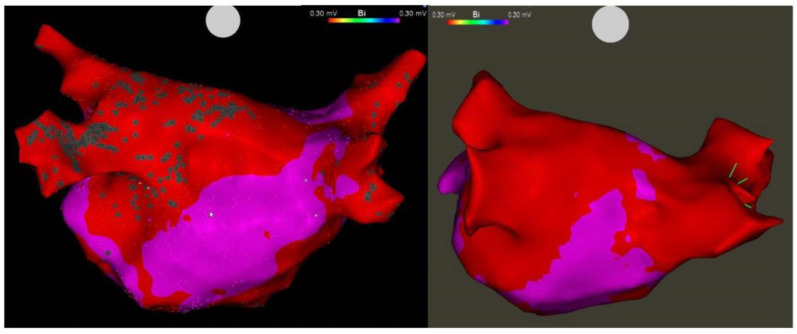
Effects of VoM ethanol infusion on posterior wall, with extensive low voltage seen both panels (right sided veins already isolated in right panel).

The technique for VoM ethanol ablation is now well established. The protocol employed in our centre has been previously described [57] and reflects a standardised approach for VoM cannulation and ethanol delivery [46,61]. We perform VoM ethanolisation for first-time or repeat persistent AF ablation. This is performed as a first-step in the procedure, as evidence suggests preceding RF ablation within the CS may result in lower rates of VoM visualisation [57]. The CS is cannulated via the femoral vein using a steerable sheath (Agilis NxT steerable introducer, Abbott). A left internal mammary artery (IMA) angioplasty catheter is advanced through the Agilis sheath into the distal CS, with the tip facing superiorly. Contrast injection via the IMA catheter while withdrawing the catheter proximally in the CS allows for identification of the VoM (Figure 1). Additional right anterior oblique (RAO) views may be helpful in the case of difficulty in identifying the VoM. The valve of Vieussens may be a useful landmark and may be seen within 1 cm distal to the VoM [62,63] in up to 63% of cases [64]. Once the VoM is identified, an angioplasty balloon (Sprinter, 2.0 × 6 mm; Medtronic - Minneapolis, MN, USA) is advanced over a wire (Marvel guide wire, Boston Scientific- Marlborough, MA, USA) and inflated to 2–3 atm to occlude the VoM. Contrast injections through the balloon are used to confirm occlusion and the need for repositioning in the case of leak. Once satisfactory occlusion is achieved, 3 × 3 mL infusions of 98% ethanol over 1 min are administered into the occluded VoM, with contrast injections in between infusions to ensure the integrity of the VoM. Once completed, a voltage map of the LA is made for assessment of the resultant scar to guide the placement of endocardial lesions to achieve mitral isthmus block (Figure 2 and Figure 3). Further additional lesions within the coronary sinus may also be required to achieve block, both on the ‘anchored wall’ facing the left atrium, and, in case of persistent failure to achieve block, on the free wall [65]. 

### 5.1. Complications and Pitfalls of VoM Ethanol Infusion

A large single centre study of 713 consecutive patients treated with VoM ethanol infusion reports a high feasibility rate with successful ethanol delivery in 88% of patients after a first attempt. In this study, failure of VoM infusion was attributable to non-identification or non-cannulation of the VoM, infusion of ethanol in the wrong vein and CS dissection or VoM perforation. A smaller area of VoM-related scarring post successful infusion was noted in the setting of a VoM with no branches, VoM dissection and mechanical leakage. The latter two situations were seen less frequently with increasing operator experience and can be overcome by avoidance of overinflation of the balloon and too-distal advancement of the wire in the case of dissection, and distal repositioning of the balloon in the case of leakage. Regarding complications in this series, 14 patients (2%) had a serious complication in line with complication rates for AF ablation in general [66]. Cardiac tamponade occurred in 1%, this tended to occur late and was serous in nature suggesting an inflammatory reaction secondary to ethanol extravasation into the pericardial space [64]. This led the authors to conclude that, in cases of VoM, perforation anti-inflammatory drugs and follow-up echocardiography should be considered. Anaphylactic shock occurred in 3 patients which should prompt a high index of suspicion of this occurrence in the event of unexplained haemodynamic collapse. 

A 2019 study of patient-related complications post VoM ethanol infusion reported a relatively high rate of contrast extravasation into the pericardial space, at 22.7% of 88 patients studied [67]. All these related from capillary rupture during infusion and were associated with trivial pericardial effusion not requiring intervention, except in the case of two patients who developed more significant effusions. A subsequent 2023 report described a case of very delayed pericarditis with significant effusion 6 months post VoM ethanolisation, highlighting the need for rigorous follow-up for at least 6 months post ablation [68]. 

A 2022 study reported on the single centre experience with VoM infusion over a 3-year period [69]. This study reports a feasibility rate of 90% and highlights the learning curve associated with the technique with an increase in success rates with reduction in procedure and fluoroscopy times over the study period. They noted a relatively high rate of delayed tamponade at 3.1%. 

In contrast, a 2019 comparative study of 254 patients undergoing AF ablation reported similar complication rates between those undergoing VOM ethanol infusion in addition to PVI versus those not [47]. A 2021 metanalysis of 10 studies including 1322 patients reported a feasibility rate of 86.7% [70]. Again, pericardial effusion and tamponade were the most frequently observed complications, with 9 tamponades in 644 patients. Nevertheless, in line with the above-mentioned comparative study, the safety profile was not significantly different in those receiving VoM ethanolisation as adjunct to catheter ablation vs. ablation alone. 

### 5.2. Potential Indications for VoM Ethanol Infusion

In our centre, VoM ethanol infusion is performed as part of a mitral linear lesion set in the following settings:

1. For first-time persistent AF ablation if an advanced AF substrate is suspected, as per the criteria employed in the Close Maze study (left atrial diameter > 45 mm, left atrial volume > 100 mls or non-self-terminating AF) [3]
2. At redo AF ablation (a) for recurrent persistent AF or (b) for recurrent paroxysmal AF when the pulmonary veins remain isolated (as part of the ongoing VEIN-AF randomised trial lesion set)
3. For atrial tachycardia involving the mitral isthmus

In all cases, VoM ethanol infusion is never performed without also completing mitral isthmus block. 

## 6. What Next: Challenges to VoM Ethanol Infusion

VoM ethanol ablation has become increasingly employed worldwide, with favourable signals regarding longer term outcomes for persistent AF, a traditionally challenging group in whom various adjunct strategies have repeatedly disappointed in the randomised setting. It is important to note, however, that while its feasibility outside high volume settings has been demonstrated, the learning curve and the possibility of higher complication rates is an important consideration when choosing to adopt this technique [71]. Moreover, considerably lengthy procedure times with a comprehensive VoM strategy (often in excess of 4 h) and significantly higher fluoroscopy doses should not be discounted [51]. 

A potential challenge to the future of the strategy may take the form of pulsed field ablation (PFA). This novel non-thermal form of energy delivery has demonstrated promise for fast and effective lesion creation for PVI, with perhaps the greatest potential benefit related to its excellent safety profile regarding collateral tissue injury [72,73,74,75]. More recently, adjunct posterior wall ablation and linear lesion sets at the roof and mitral isthmus have proven feasible and fast, using PFA with high rates of durability in small studies [76,77,78]. Should larger studies continue to support a role for PFA in the creation of linear lesions, particularly at the mitral ithmus, with an enhanced safety profile and shorter procedure times on its side, it would call into question the future of VOM ethanolisation. Nevertheless, the ability of PFA to adequately ablate the epicardial structures often responsible for perpetuation of AF and residual conduction across isthmi has yet to be established. Further randomised controlled trials are needed to answer these important questions.

## 7. Conclusions

VoM ethanol infusion is a promising technique that has shown consistent benefit in the achievement of durable mitral isthmus block, a considerable challenge when performing linear ablation in this difficult region. This durability has translated into promising early data emerging from studies incorporating the technique into an extensive endpoint driven lesion set for persistent AF patients and may also relate to autonomic effects and elimination of AF triggers. The technical complexities of the technique, with longer procedure and fluoroscopy times, merit consideration and, in parallel, the performance of PFA for linear ablation at the mitral isthmus may present a challenge to the future viability of the technique. Further randomised controlled trials are needed before advocation for widespread use.

## References

[1] Krijthe B.P., Kunst A., Benjamin E.J., Lip G.Y.H., Franco O.H., Hofman A., Witteman J.C.M., Stricker B.H., Heeringa J. (2013). Projections on the number of individuals with atrial fibrillation in the European Union, from 2000 to 2060. Eur. Heart J..

[2] Duytschaever M., De Pooter J., Demolder A., El Haddad M., Phlips T., Strisciuglio T., Debonnaire P., Wolf M., Vandekerckhove Y., Knecht S. (2020). Long-term impact of catheter ablation on arrhythmia burden in low-risk patients with paroxysmal atrial fibrillation: The CLOSE to CURE study. Heart Rhythm.

[3] O’Neill L., Almorad A., El Haddad M., Wielandts J.-Y., Gillis K., Hilfiker G., de Becker B., Lycke M., Tavernier R., le Polain de Waroux J.-B. (2023). Impact of Catheter Ablation on Arrhythmia Burden in Patients With Shock-Resistant Persistent Atrial Fibrillation. JACC Clin. Electrophysiol..

[4] Chew D.S., Li Z., Steinberg B.A., O’Brien E.C., Pritchard J., Bunch T.J., Mark D.B., Patel M.R., Nabutovsky Y., Greiner M.A. (2022). Arrhythmic Burden and the Risk of Cardiovascular Outcomes in Patients With Paroxysmal Atrial Fibrillation and Cardiac Implanted Electronic Devices. Circ. Arrhythmia Electrophysiol..

[5] Glotzer T.V., Daoud E.G., Wyse D.G., Singer D.E., Ezekowitz M.D., Hilker C., Miller C., Qi D., Ziegler P.D. (2009). The Relationship between daily atrial tachyarrhythmia burden from implantable device diagnostics and stroke risk the trends study. Circ. Arrhythmia Electrophysiol..

[6] Brachmann J., Sohns C., Andresen D., Siebels J., Sehner S., Boersma L., Merkely B., Pokushalov E., Sanders P., Schunkert H. (2021). Atrial Fibrillation Burden and Clinical Outcomes in Heart Failure: The CASTLE-AF Trial. JACC Clin. Electrophysiol..

[7] Taghji P., El Haddad M., Phlips T., Wolf M., Knecht S., Vandekerckhove Y., Tavernier R., Nakagawa H., Duytschaever M. (2018). Evaluation of a Strategy Aiming to Enclose the Pulmonary Veins With Contiguous and Optimized Radiofrequency Lesions in Paroxysmal Atrial Fibrillation: A Pilot Study. JACC Clin. Electrophysiol..

[8] Mont L., Bisbal F., Hernandez-Madrid A., Perez-Castellano N., Vinolas X., Arenal A., Arribas F., Fernandez-Lozano I., Bodegas A., Cobos A. (2014). Catheter ablation vs. antiarrhythmic drug treatment of persistent atrial fibrillation: A multicentre, randomized, controlled trial (SARA study). Eur. Heart J..

[9] Verma A., Jiang C., Betts T.R., Chen J., Deisenhofer I., Mantovan R., Macle L., Morillo C.A., Haverkamp W., Weerasooriya R. (2015). Approaches to Catheter Ablation for Persistent Atrial Fibrillation. N. Engl. J. Med..

[10] Kistler P.M., Chieng D., Sugumar H., Ling L.-H., Segan L., Azzopardi S., Al-Kaisey A., Parameswaran R., Anderson R.D., Hawson J. (2023). Effect of Catheter Ablation Using Pulmonary Vein Isolation With vs. Without Posterior Left Atrial Wall Isolation on Atrial Arrhythmia Recurrence in Patients With Persistent Atrial Fibrillation: The CAPLA Randomized Clinical Trial. JAMA.

[11] Huo Y., Gaspar T., Schönbauer R., Wójcik M., Fiedler L., Roithinger F.X., Martinek M., Pürerfellner H., Kirstein B., Richter U. (2022). Low-Voltage Myocardium-Guided Ablation Trial of Persistent Atrial Fibrillation. NEJM Evid..

[12] Marrouche N.F., Wazni O., McGann C., Greene T., Dean J.M., Dagher L., Kholmovski E., Mansour M., Marchlinski F., Wilber D. (2022). Effect of MRI-Guided Fibrosis Ablation vs. Conventional Catheter Ablation on Atrial Arrhythmia Recurrence in Patients With Persistent Atrial Fibrillation: The DECAAF II Randomized Clinical Trial. JAMA.

[13] Narayan S.M., Krummen D.E., Shivkumar K., Clopton P., Rappel W.J., Miller J.M. (2012). Treatment of atrial fibrillation by the ablation of localized sources: CONFIRM (Conventional Ablation for Atrial Fibrillation With or Without Focal Impulse and Rotor Modulation) trial. J. Am. Coll. Cardiol..

[14] Nademanee K., McKenzie J., Kosar E., Schwab M., Sunsaneewitayakul B., Vasavakul T., Khunnawat C., Ngarmukos T. (2004). A new approach for catheter ablation of atrial fibrillation: Mapping of the electrophysiologic substrate. J. Am. Coll. Cardiol..

[15] Kamanu S., Tan A.Y., Peter C.T., Hwang C., Chen P.S. (2006). Vein of Marshall activity during sustained atrial fibrillation. J. Cardiovasc. Electrophysiol..

[16] Ulphani J.S., Arora R., Cain J.H., Villuendas R., Shen S., Gordon D., Inderyas F., Harvey L.A., Morris A., Goldberger J.J. (2007). The Ligament of Marshall as a Parasympathetic Conduit. Am. J. Physiol.-Heart Circ. Physiol..

[17] Ge W., Li T., Lu Y., Jiang J., Tung T., Yan S. (2023). Efficacy and feasibility of vein of Marshall ethanol infusion during persistent atrial fibrillation ablation: A systematic review and meta-analysis. Clin. Cardiol..

[18] Valderrábano M., Peterson L.E., Swarup V., Schurmann P.A., Makkar A., Doshi R.N., Delurgio D., Athill C.A., Ellenbogen K.A., Natale A. (2020). Effect of Catheter Ablation with Vein of Marshall Ethanol Infusion vs. Catheter Ablation Alone on Persistent Atrial Fibrillation: The VENUS Randomized Clinical Trial. JAMA-J. Am. Med. Assoc..

[19] VI On The Development of the Great Anterior Veins in Man and Mammalia; Including an Account of Certain Remnants of Fœtal Structure Found in the Adult, a Comparative View of These Great Veins the Different Mammalia, and an Analysis of Their Occasional Peculiarities in the Human Subject|Philosophical Transactions of the Royal Society of London. https://royalsocietypublishing.org/doi/10.1098/rstl.1850.0007.

[20] Rodríguez-Mañero M., Schurmann P., Valderrábano M. (2016). Ligament and vein of Marshall: A therapeutic opportunity in atrial fibrillation. Heart Rhythm.

[21] Gilard M., Mansourati J., Etienne Y., Larlet J.M., Truong B., Boschat J., Blanc J.J. (1998). Angiographic anatomy of the coronary sinus and its tributaries. Pacing Clin. Electrophysiol. PACE.

[22] Hwang C., Chen P.-S. (2009). Ligament of Marshall: Why it is important for atrial fibrillation ablation. Heart Rhythm.

[23] Makino M., Inoue S., Matsuyama T.-A., Ogawa G., Sakai T., Kobayashi Y.-I., Katagiri T., Ota H. (2006). Diverse myocardial extension and autonomic innervation on ligament of Marshall in humans. J. Cardiovasc. Electrophysiol..

[24] Han S., Joung B., Scanavacca M., Sosa E., Chen P.-S., Hwang C. (2010). Electrophysiological characteristics of the Marshall bundle in humans. Heart Rhythm.

[25] Tan A.Y., Chou C.C., Zhou S., Nihei M., Hwang C., Peter C.T., Fishbein M.C., Chen P.S. (2006). Electrical connections between left superior pulmonary vein, left atrium, and ligament of Marshall: Implications for mechanisms of atrial fibrillation. Am. J. Physiol.-Heart Circ. Physiol..

[26] Inferior Interatrial Pathway in the Dog—PubMed. https://pubmed.ncbi.nlm.nih.gov/5038734/.

[27] Kim D.T., Lai A.C., Hwang C., Fan L.T., Karagueuzian H.S., Chen P.S., Fishbein M.C. (2000). The ligament of Marshall: A structural analysis in human hearts with implications for atrial arrhythmias. J. Am. Coll. Cardiol..

[28] Lin J., Scherlag B.J., Lu Z., Zhang Y., Liu S., Patterson E., Jackman W.M., Lazzara R., Po S.S. (2008). Inducibility of Atrial and Ventricular Arrhythmias Along the Ligament of Marshall: Role of Autonomic Factors. J. Cardiovasc. Electrophysiol..

[29] Báez-Escudero J.L., Keida T., Dave A.S., Okishige K., Valderrábano M. (2014). Ethanol infusion in the vein of Marshall leads to parasympathetic denervation of the human left atrium: Implications for atrial fibrillation. J. Am. Coll. Cardiol..

[30] Doshi R.N., Wu T.J., Yashima M., Kim Y.H., Ong J.J., Cao J.M., Hwang C., Yashar P., Fishbein M.C., Karagueuzian H.S. (1999). Relation between ligament of Marshall and adrenergic atrial tachyarrhythmia. Circulation.

[31] Hwang C., Karagueuzian H.S., Chen P.S. (1999). Idiopathic paroxysmal atrial fibrillation induced by a focal discharge mechanism in the left superior pulmonary vein: Possible roles of the ligament of Marshall. J. Cardiovasc. Electrophysiol..

[32] Hwang C., Wu T.J., Doshi R.N., Peter C.T., Chen P.S. (2000). Vein of marshall cannulation for the analysis of electrical activity in patients with focal atrial fibrillation. Circulation.

[33] O’Neill L., Duytschaever M., Le Polain De Waroux J.-B., Konrad T., Rostock T., Derval N., Pambrun T., Rollin A., Maury P., Knecht S. (2022). Noninducibility as an Ablation Strategy for Atrial Tachycardia After First-Time Persistent AF Ablation. JACC Clin. Electrophysiol..

[34] Chae S., Oral H., Good E., Dey S., Wimmer A., Crawford T., Wells D., Sarrazin J.F., Chalfoun N., Kuhne M. (2007). Atrial Tachycardia After Circumferential Pulmonary Vein Ablation of Atrial Fibrillation. Mechanistic Insights, Results of Catheter Ablation, and Risk Factors for Recurrence. J. Am. Coll. Cardiol..

[35] Morita H., Zipes D.P., Morita S.T., Wu J. (2012). The role of coronary sinus musculature in the induction of atrial fibrillation. Heart Rhythm.

[36] Vlachos K., Denis A., Kitamura T., Takigawa M., Frontera A., Martin R., Bourier F., Martin C., Cheniti G., Pambrun T. (2021). The role of marshall bundle epicardial connections in atrial tachycardias after atrial fibrillation ablation. EP Eur..

[37] Vlachos K., Derval N., Pambrun T., Duchateau J., Martin C.A., Bazoukis G., Frontera A., Takigawa M., Nakashima T., Efremidis M. (2021). Ligament of Marshall ablation for persistent atrial fibrillation. Pacing Clin. Electrophysiol..

[38] Hwang C., Fishbein M.C., Chen P.-S. (2006). How and when to ablate the ligament of Marshall. Heart Rhythm.

[39] Kashimura S., Fujisawa T., Nakajima K., Kunitomi A., Katsumata Y., Nishiyama T., Kimura T., Nishiyama N., Aizawa Y., Fukuda K. (2020). Electrical Isolation of the Marshall Bundle by Radiofrequency Catheter Ablation: In Patients With Atrial Fibrillation. JACC Clin. Electrophysiol..

[40] Becker A.E. (2004). Left atrial isthmus: Anatomic aspects relevant for linear catheter ablation procedures in humans. J. Cardiovasc. Electrophysiol..

[41] Latcu D.G., Squara F., Massaad Y., Bun S.S., Saoudi N., Marchlinski F.E. (2016). Electroanatomic characteristics of the mitral isthmus associated with successful mitral isthmus ablation. Europace.

[42] Yokokawa M., Sundaram B., Garg A., Stojanovska J., Oral H., Morady F., Chugh A. (2011). Impact of mitral isthmus anatomy on the likelihood of achieving linear block in patients undergoing catheter ablation of persistent atrial fibrillation. Heart Rhythm.

[43] Valderrábano M., Chen H.R., Sidhu J., Rao L., Ling Y., Khoury D.S. (2009). Retrograde ethanol infusion in the vein of Marshall: Regional left atrial ablation, vagal denervation and feasibility in humans. Circ. Arrhythmia Electrophysiol..

[44] Valderrábano M., Liu X., Sasaridis C., Sidhu J., Little S., Khoury D.S. (2009). Ethanol Infusion in the Vein of Marshall: Adjunctive Effects during Ablation of Atrial Fibrillation. Heart Rhythm Off. J. Heart Rhythm Soc..

[45] Huang L., Gao M., Lai Y., Guo Q., Li S., Li C., Liu N., Wang W., Liu X., Zuo S. (2023). The adjunctive effect for left pulmonary vein isolation of vein of Marshall ethanol infusion in persistent atrial fibrillation. EP Europace.

[46] Dave A.S., Báez-Escudero J.L., Sasaridis C., Hong T.E., Rami T., Valderrábano M. (2012). Role of the vein of Marshall in atrial fibrillation recurrences after catheter ablation: Therapeutic effect of ethanol infusion. J. Cardiovasc. Electrophysiol..

[47] Liu C.-M., Lo L.-W., Lin Y.-J., Lin C.-Y., Chang S.-L., Chung F.-P., Chao T.-F., Hu Y.-F., Tuan T.-C., Liao J.-N. (2019). Long-term efficacy and safety of adjunctive ethanol infusion into the vein of Marshall during catheter ablation for nonparoxysmal atrial fibrillation. J. Cardiovasc. Electrophysiol..

[48] Lador A., Peterson L.E., Swarup V., Schurmann P.A., Makkar A., Doshi R.N., DeLurgio D., Athill C.A., Ellenbogen K.A., Natale A. (2021). Determinants of Outcome Impact of Vein of Marshall Ethanol Infusion When Added to Catheter Ablation of Persistent Atrial Fibrillation: A Secondary Analysis of the VENUS Randomized Clinical Trial. Heart Rhythm.

[49] Li F., Sun J.-Y., Wu L.-D., Zhang L., Qu Q., Wang C., Qian L.-L., Wang R.-X. (2022). The Long-Term Outcomes of Ablation with Vein of Marshall Ethanol Infusion vs. Ablation Alone in Patients With Atrial Fibrillation: A Meta-Analysis. Front. Cardiovasc. Med..

[50] Pambrun T., Denis A., Duchateau J., Sacher F., Hocini M., Jaïs P., Haïssaguerre M., Derval N. (2019). MARSHALL bundles elimination, Pulmonary veins isolation and Lines completion for ANatomical ablation of persistent atrial fibrillation: MARSHALL-PLAN case series. J. Cardiovasc. Electrophysiol..

[51] Derval N., Duchateau J., Denis A., Ramirez F.D., Mahida S., André C., Krisai P., Nakatani Y., Kitamura T., Takigawa M. (2021). Marshall bundle elimination, Pulmonary vein isolation, and Line completion for ANatomical ablation of persistent atrial fibrillation (Marshall-PLAN): Prospective, single-center study. Heart Rhythm.

[52] Lai Y., Liu X., Sang C., Long D., Li M., Ge W., Liu X., Lu Z., Guo Q., Jiang C. (2021). Effectiveness of Ethanol Infusion into the Vein of Marshall Combined with a Fixed Anatomical Ablation Strategy (the “Upgraded 2C3L” Approach) for Catheter Ablation of Persistent Atrial Fibrillation. J. Cardiovasc. Electrophysiol..

[53] Novel Ablation Strategy Improves Freedom from Arrhythmias in Atrial Fibrillation Patients. https://www.escardio.org/The-ESC/Press-Office/Press-releases/novel-ablation-strategy-improves-freedom-from-arrhythmias-in-atrial-fibrillation.

[54] Liu X.-X., Liu Q., Lai Y.-W., Guo Q., Bai R., Long D.-Y., Yu R.-H., Tang R.-B., Liu N., Jiang C.-X. (2023). Prospective randomized comparison between upgraded ‘2C3L’ vs. PVI approach for catheter ablation of persistent atrial fibrillation: PROMPT-AF trial design. Am. Heart J..

[55] Nakashima T., Pambrun T., Vlachos K., Goujeau C., André C., Krisai P., Ramirez F.D., Kamakura T., Takagi T., Nakatani Y. (2020). Impact of Vein of Marshall Ethanol Infusion on Mitral Isthmus Block: Efficacy and Durability. Circ. Arrhythm. Electrophysiol..

[56] Takigawa M., Vlachos K., Martin C.A., Bourier F., Denis A., Kitamura T., Cheniti G., Lam A., Martin R., Frontera A. (2020). Acute and Mid-Term Outcome of Ethanol Infusion of Vein of Marshall for the Treatment of Perimitral Flutter. EP Eur..

[57] Gillis K., O’Neill L., Wielandts J.-Y., Hilfiker G., Almorad A., Lycke M., El Haddad M., le Polain de Waroux J.-B., Tavernier R., Duytschaever M. (2022). Vein of Marshall Ethanol Infusion as First Step for Mitral Isthmus Linear Ablation. JACC Clin. Electrophysiol..

[58] Du X., Luo C., Shen C., Xu Y., Feng M., Jin H., Fu G., Wang B., Liu J., Gao F. (2023). The impact of empirical Marshall vein ethanol infusion as a first-choice intraoperative strategy on the long-term outcomes in patients with persistent atrial fibrillation undergoing mitral isthmus ablation. Front. Cardiovasc. Med..

[59] Ascione C., Kowalewski C., Pambrun T., Derval N., Jaïs P. (2023). A Posterior Wall Resistant to Electroporation Finally Blocked With Vein of Marshall Ethanol Infusion. JACC Clin. Electrophysiol..

[60] Ishimura M., Yamamoto M., Himi T., Kobayashi Y. (2023). Efficacy and durability of posterior wall isolation with ethanol infusion into the vein of Marshall. J. Cardiovasc. Electrophysiol..

[61] Báez-Escudero J.L., Morales P.F., Dave A.S., Sasaridis C.M., Kim Y.H., Okishige K., Valderrábano M. (2012). Ethanol infusion in the vein of Marshall facilitates mitral isthmus ablation. Heart Rhythm.

[62] de Oliveira I.M., Scanavacca M.I., Correia A.T., Sosa E.A., Aiello V.D. (2007). Anatomic relations of the Marshall vein: Importance for catheterization of the coronary sinus in ablation procedures. EP Eur..

[63] Von Lüudinghausen M., Ohmachi N., Besch S., Mettenleiter R. (1995). Atrial veins of the human heart. Clin. Anat..

[64] Kamakura T., Derval N., Duchateau J., Denis A., Nakashima T., Takagi T., Ramirez F.D., André C., Krisai P., Nakatani Y. (2021). Vein of Marshall Ethanol Infusion: Feasibility, Pitfalls, and Complications in Over 700 Patients. Circ. Arrhythm. Electrophysiol..

[65] Pambrun T., Derval N., Duchateau J., Denis A., Chauvel R., Tixier R., Welte N., André C., Nakashima T., Nakatani Y. (2021). Epicardial course of the musculature related to the great cardiac vein: Anatomical considerations and clinical implications for mitral isthmus block after vein of Marshall ethanol infusion. Heart Rhythm.

[66] Gupta A., Perera T., Ganesan A., Sullivan T., Lau D.H., Roberts-Thomson K.C., Brooks A.G., Sanders P. (2013). Complications of catheter ablation of atrial fibrillation: A systematic review. Circ. Arrhythm. Electrophysiol..

[67] Kato K., Tanaka A., Morimoto S., Hasegawa S., Ishiguro N., Kametani R., Hattori H., Shibata N. (2019). Potential complications in patients undergoing an ethanol injection into the vein of Marshall. J. Cardiovasc. Electrophysiol..

[68] Motoki K., Harada T., Hosokawa S., Hara T., Yamamoto K., Kishi K. (2023). Very delayed pericarditis associated with ethanol ablation of the vein of Marshall for treatment of atrial fibrillation. Hear. Case Rep..

[69] Leyton-Mange J.S., Tandon K., Sze E.Y., Carpenter C.M., Sesselberg H.W. (2023). The Maine vein of Marshall ethanol experience: Learning curve and safety. J. Interv. Card. Electrophysiol. Int. J. Arrhythm. Pacing.

[70] He Z., Yang L., Bai M., Yao Y., Zhang Z. (2021). Feasibility, efficacy, and safety of ethanol infusion into the vein of Marshall for atrial fibrillation: A meta-analysis. Pacing Clin. Electrophysiol. PACE.

[71] Knecht S., Sticherling C. (2023). Ethanol infusion in the vein of Marshall is feasible in experienced centers—But is it ready for every Tom, Dick, and Harry?. J. Interv. Card. Electrophysiol..

[72] Reddy V.Y., Neuzil P., Koruth J.S., Petru J., Funosako M., Cochet H., Sediva L., Chovanec M., Dukkipati S.R., Jais P. (2019). Pulsed Field Ablation for Pulmonary Vein Isolation in Atrial Fibrillation. J. Am. Coll. Cardiol..

[73] Reddy V.Y., Dukkipati S.R., Neuzil P., Anic A., Petru J., Funasako M., Cochet H., Minami K., Breskovic T., Sikiric I. (2021). Pulsed Field Ablation of Paroxysmal Atrial Fibrillation: 1-Year Outcomes of IMPULSE, PEFCAT, and PEFCAT II. JACC Clin. Electrophysiol..

[74] Duytschaever M., De Potter T., Grimaldi M., Anic A., Vijgen J., Neuzil P., Van Herendael H., Verma A., Skanes A., Scherr D. (2023). Paroxysmal Atrial Fibrillation Ablation Using a Novel Variable-Loop Biphasic Pulsed Field Ablation Catheter Integrated With a 3-Dimensional Mapping System: 1-Year Outcomes of the Multicenter inspIRE Study. Circ. Arrhythmia Electrophysiol..

[75] Verma A., Haines D.E., Boersma L.V., Sood N., Natale A., Marchlinski F.E., Calkins H., Sanders P., Packer D.L., Kuck K.-H. (2023). Pulsed Field Ablation for the Treatment of Atrial Fibrillation: PULSED AF Pivotal Trial. Circulation.

[76] Reddy V.Y., Anic A., Koruth J., Petru J., Funasako M., Minami K., Breskovic T., Sikiric I., Dukkipati S.R., Kawamura I. (2020). Pulsed Field Ablation in Patients With Persistent Atrial Fibrillation. J. Am. Coll. Cardiol..

[77] Reddy V.Y., Anter E., Rackauskas G., Peichl P., Koruth J.S., Petru J., Funasako M., Minami K., Natale A., Jais P. (2020). Lattice-Tip Focal Ablation Catheter That Toggles between Radiofrequency and Pulsed Field Energy to Treat Atrial Fibrillation: A First-in-Human Trial. Circ. Arrhythmia Electrophysiol..

[78] Reddy V.Y., Peichl P., Anter E., Rackauskas G., Petru J., Funasako M., Minami K., Koruth J.S., Natale A., Jais P. (2023). A Focal Ablation Catheter Toggling Between Radiofrequency and Pulsed Field Energy to Treat Atrial Fibrillation. JACC Clin. Electrophysiol..

